# *SERPINA1* PiZ and PiS Heterozygotes and Lung Function Decline in the SAPALDIA Cohort

**DOI:** 10.1371/journal.pone.0042728

**Published:** 2012-08-13

**Authors:** Gian-Andri Thun, Ilaria Ferrarotti, Medea Imboden, Thierry Rochat, Margaret Gerbase, Florian Kronenberg, Pierre-Olivier Bridevaux, Elisabeth Zemp, Michele Zorzetto, Stefania Ottaviani, Erich W. Russi, Maurizio Luisetti, Nicole M. Probst-Hensch

**Affiliations:** 1 Swiss Tropical and Public Health Institute, Basel, Switzerland; 2 University of Basel, Basel, Switzerland; 3 Center for Diagnosis of Inherited Alpha1-antitrypsin Deficiency, Institute for Respiratory Disease, IRCCS San Matteo Hospital Foundation, University of Pavia, Pavia, Italy; 4 Division of Pulmonary Medicine, University Hospital of Geneva, Geneva, Switzerland; 5 Division of Genetic Epidemiology, Department of Medical Genetics, Molecular and Clinical Pharmacology, Innsbruck Medical University, Innsbruck, Austria; 6 Pulmonary Division, University Hospital of Zurich, Zurich, Switzerland; Clinica Universidad de Navarra, Spain

## Abstract

**Background:**

Severe alpha1-antitrypsin (AAT) deficiency is a strong risk factor for COPD. But the impact of gene variants resulting in mild or intermediate AAT deficiency on the longitudinal course of respiratory health remains controversial. There is indication from experimental studies that pro-inflammatory agents like cigarette smoke can interact with these variants and thus increase the risk of adverse respiratory health effects. Therefore, we tested the effect of the presence of a protease inhibitor (Pi) S or Z allele (PiMS and PiMZ) on the change in lung function in different inflammation-exposed subgroups of a large, population-based cohort study.

**Methodology and Principal Findings:**

The SAPALDIA population includes over 4600 subjects from whom *SERPINA1* genotypes for S and Z alleles, spirometry and respiratory symptoms at baseline and after 11 years follow-up, as well as proxies for inflammatory conditions, such as detailed smoking history, obesity and high sensitivity C-reactive protein (hs-CRP), were available. All analyses were performed by applying multivariate regression models. There was no overall unfavourable effect of PiMS or PiMZ genotype on lung function change. We found indication that PiZ heterozygosity interacted with inflammatory stimuli leading to an accelerated decline in measures in use as indices for assessing mild airway obstruction. Obese individuals with genotype PiMM had an average annual decline in the forced mid expiratory flow (**Δ**FEF25-75%) of 58.4 ml whereas in obese individuals with PiMZ it amounted to 92.2 ml (p = 0.03). Corresponding numbers for persistent smokers differed even more strongly (66.8 ml (PiMM) vs. 108.2 ml (PiMZ), p = 0.005). Equivalent, but less strong associations were observed for the change in the FEV1/FVC ratio.

**Conclusions:**

We suggest that, in addition to the well established impact of the rare PiZZ genotype, one Z allele may be sufficient to accelerate lung function decline in population subgroups characterized by elevated levels of low grade inflammation.

## Introduction

Reduced lung function measures are the most common diagnostic parameter to detect airway obstruction which is the main characteristic of asthma and chronic obstructive pulmonary disease (COPD). The only functionally characterized gene variants associated with airway obstruction and increased loss of lung function are infrequent polymorphisms in the *SERPINA1* gene causing deficiency of alpha1-antitrypsin (AAT) [1]. This antiprotease inhibits neutrophil elastase, an enzyme that degrades pulmonary elastic fibers. Homozygosity for the protease inhibitor deficiency variant Z (PiZZ, also referred to as severe AAT deficiency) and compound heterozygosity for both deficiency variants S and Z (PiSZ) are widely accepted risk factors for airway obstruction and accelerated lung function decline, particularly among smokers [2]. But since these allele combinations all have frequencies below 0.1% in the general European population [3], they only account for 2-5% of all COPD cases. The more prevalent heterozygous genotypes PiMS and PiMZ (M stands for the wildtype allele) reduce the AAT blood levels only slightly [4] and are therefore referred to as mild (for PiMS) and intermediate (for PiMZ) AAT deficiencies.

While PiMS is generally believed not to be associated with low lung function or a higher risk of COPD [5], the evidence for PiMZ remains unclear even in the light of a meta-analysis [6]. The few population-based longitudinal studies have not shown adverse health effects, but they varied with regard to the phenotype studied and the inclusion of gene-environment interactions [7], [8], [9]. An investigation restricted to smokers showed that PiMZ was overrepresented in the group with rapid FEV1 (forced expiratory volume in one second) decline, suggesting that susceptibility may be refined to population subgroups with elevated inflammatory and proteolytic stress in the lungs [10]. These processes may locally increase cleavage, as well as oxidant-induced inactivation [11] and polymerization [12] of AAT, leading to a further reduction of this enzyme in PiMZ carriers. Apart from inhalant triggers like smoking, systemic inflammation may also compromise pulmonary health [13], [14] and therefore particularly affect individuals with reduced AAT levels.

We hypothesized therefore that the intrapulmonary anti-proteolytic capacity in people with mild or intermediate AAT deficiency may not be sufficient to counterbalance an excess of inflammatory triggers targeting the respiratory system. We used the SAPALDIA cohort (Swiss Cohort Study on Air Pollution and Lung and Heart Diseases in Adults) to test such possibly unfavourable effects of PiS or PiZ heterozygosity on the longitudinal course of lung function over 11 years of follow-up in the general population. The large and well characterized study population allowed us to particularly study subgroups exposed to elevated local airway or systemic inflammatory conditions, such as active and passive smokers, and people suffering from obesity.

## Results

### Study Sample

A comparison of characteristics between SAPALDIA follow-up participants included and excluded in this study is provided in Table 1. The study population consisted of a healthier sample with a higher percentage of never-smokers and fewer obese individuals. A comparison between the three genotype classes showed highly significant differences in AAT serum levels (Table 1). Compared to PiM homozygotes, unadjusted AAT serum concentrations were more than 16% and 38% reduced in PiS and PiZ heterozygotes, respectively. There were no differences in circulating high-sensitivity C-reactive protein (hs-CRP), the main marker for systemic inflammation, between the genotype groups. Furthermore, the genotype classes differed slightly in the unadjusted forced expiratory flow over the middle half of FVC (FEF25-75%) at baseline and in unadjusted declines of FEF25-75%.

**Table 1:** Characteristics of SAPALDIA follow-up participants at baseline (1991; upper part), follow-up (2002; middle part), and between the two examinations (lower part).

	Not included in Study (N = 3372)	Included in Study (N = 4675)	PiMM (N = 4207)	PiMS (N = 356)	PiMZ (N = 112)	P-value^a^
***At baseline***						
% women	51.2	52.4	52.6	50.8	53.6	0.80
Age (y), median, IQR	42.4 (32.6, 51.1)	41.4 (31.7, 49.7)	41.5 (31.6, 49.8)	41.3 (32.9, 49.6)	38.3 (31.3, 47.3)	0.33
% never smokers	39.9 (N = 3360)	49.6	49.3	51.7	54.5	0.39
% current smokers	34.9 (N = 3360)	29.6	30.0	27.2	24.1	0.24
% ETS in never smokers	30.6 (N = 1340)	28.3 (N = 2318)	28.5 (N = 2073)	24.5 (N = 184)	34.4 (N = 61)	0.29
BMI (kg/m^2^), median, IQR	23.7 (21.3, 26.3; N = 3306)	23.1 (21.1, 25.6)	23.1 (21.0, 25.7)	23.3 (21.2, 25.7)	22.9 (21.3, 24.9)	0.80
% obese^b^	7.6 (N = 3306)	5.3	5.4	4.5	4.5	0.72
FEV1 (mL), mean±SD	3485±864 (N = 2910)	3576±824	3570±825	3617±819	3627 ± 787	0.35
FVC (mL), mean±SD	4415±1062 (N = 2929)	4527±1022	4522±1025	4580±1000	4553±987	0.57
FEF25-75% (mL), mean±SD	3371±1228 (N = 2794)	3436±1223	3426±1221	3468±1212	3706±1320	0.05
FEV1/FVC (%), mean±SD	79.10±7.93 (N = 2794)	79.21±7.59	79.18±7.56	79.07±7.83	80.61±8.01	0.14
***At follow-up***						
% never smokers	40.7 (N = 2936)	48.1	47.8	49.7	53.6	0.40
% current smokers	32.3 (N = 2936)	22.6	22.8	21.9	16.1	0.23
% ETS in never smokers	15.1 (N = 1189)	15.4 (N = 2249)	15.5 (N = 2012)	14.7 (N = 177)	15.0 (N = 60)	0.96
BMI (kg/m^2^), median, IQR	25.7 (23.0, 28.8; N = 1923)	25.2 (22.7, 28.2)	25.2 (22.7, 28.2)	25.4 (22.8, 28.4)	25.2 (23.1, 27.6)	0.88
% obese^b^	18.1 (N = 1923)	15.2	15.2	15.2	14.3	0.96
AAT (g/L), mean±SD	1.25±0.22 (N = 1642)	1.27±0.20	1.30±0.19	1.09±0.16	0.80±0.11	<0.001
hs-CRP (mg/L), median, IQR	1.1 (0.5, 2.3; N = 1642)	1.0 (0.5, 2.3)	1.0 (0.5, 2.3)	1.0 (0.5, 2.0)	0.9 (0.5, 2.5)	0.16
***Between baseline and follow-up***						
**Δ**FEV1 (mL/y), mean±SD	-35.2±32.6 (N = 1278)	-35.3±29.8	-35.1±29.8	-37.7±30.1	-36.3±28.7	0.27
**Δ**FVC (mL/y), mean±SD	-26.1±43.7 (N = 1266)	-24.1±40.2	-24.1±40.2	-25.0±39.7	-22.4±41.5	0.82
**Δ**FEF25-75% (mL/y), mean±SD	-64.2±64.1 (N = 1070)	-71.1±65.2	-70.4±64.8	-76.0±64.9	-84.3±77.9	0.03
**Δ** (FEV1/FVC) (‰/y), mean±SD	-3.49±5.19 (N = 1071)	-4.05±4.95	-4.01±4.94	-4.26±5.01	-4.62±4.99	0.32

AAT: Alpha1-antitrypsin; BMI: Body mass index; ETS: regularly exposed to environmental tobacco smoke; FEF25-75%: mid expiratory flow; FEV1: forced expiratory volume in 1 second; FVC: forced vital capacity; hs-CRP: high-sensitivty C-reactive protein; IQR: Interquartile range; SD: Standard deviation.

a Pearson's χ2 test for testing equal proportions, analysis of variance (ANOVA) for testing equal means of normally distributed data (AAT levels and spirometric measurements), and Kruskal-Wallis test for testing equal distributions in not normally distributed data (age and BMI values) between the three genotype classes.

b Obese subjects were defined as having a BMI≥30kg/m^2^.

### Adjusted Spirometric Decline Rates according to Genotype

In adjusted models, neither PiMS nor PiMZ subjects exhibited statistically significant steeper annual declines than PiMM individuals in any measure of lung function (all p≥0.07, Table 2 and Table 3). Hypothesizing that PiMS and PiMZ carriers can only compensate their reduced anti-proteolytic capacity in pulmonary tissue if no excess of pulmonary or systemic inflammation is present, we tested if stratification by smoking or obesity status may alter these associations. While we could not find any significant association between the presence of a S or Z allele and **Δ**FEV1 or **Δ**FVC (forced vital capacity) irrespective of the smoking or obesity category (Table 2), smokers and obese individuals with PiMZ genotype showed elevated declines in FEF25-75% (Table 3). In ever smokers, PiMZ carriers lost on average additional 17.4 ml per year compared to PiMM in FEF25-75% (p = 0.05), and this difference became more pronounced in people who smoked at baseline and follow-up (41.4 ml in persistent smokers, p = 0.005). A similar pattern could be observed in obese participants (**Δ**FEF25-75% = 58.4 ml (PiMM) vs. 92.2 ml (PiMZ), p = 0.03). There was no such effect in never-smokers exposed to environmental tobacco smoke. Values for **Δ** (FEV1/FVC) consistently confirmed this trend, but associations did not reach statistical significance. For **Δ**FEF25-75%, the presence of a Z allele interacted statistically significant with smoking (p_interaction_ = 0.04 with smoking status (persistent vs. never) and p_interaction_ = 0.002 with packyears between the two surveys), but not with obesity status (p = 0.14). Statistically significant modification of the Z allele effect on **Δ** (FEV1/FVC) could be observed for packyears between the two surveys and obesity status (p_interaction_ = 0.04 and 0.08, respectively). As we had previously found sex differences in the association between circulating AAT concentrations and lung function [15], we further evaluated a possible effect modification by sex. However, sex did not significantly modify the allele effect on any lung function measure (all p_interaction_ ≥ 0.15).

**Table 2:** Adjusted mean values in **Δ**FEV1 and **Δ**FVC over 11 years of follow-up comparing different *SERPINA1* genotypes.

	ΔFEV1(ml/y)	95%CI	P-value	ΔFVC(ml/y)	95%CI	P-value
**Total**						
PiMM, N = 4207	-35.2	-36.0 to -34.4		-24.1	-25.2 to -23.0	
PiMS, N = 356	-36.9	-39.7 to -34.1	0.24	-24.7	-28.3 to -21.0	0.77
PiMZ, N = 112	-36.0	-41.0 to -31.1	0.74	-23.3	-29.8 to -16.7	0.80
**Passive Smokers** a						
PiMM, N = 691	-32.3	-34.5 to -30.2		-21.4	-24.1 to -18.6	
PiMS, N = 52	-33.9	-41.5 to -26.3	0.69	-26.3	-35.9 to -16.7	0.33
PiMZ, N = 22	-41.3	-52.9 to -29.7	0.14	-34.5	-49.2 to -19.7	0.09
**Ever Smokers**						
PiMM, N = 2194	-35.1	-36.4 to -33.8		-25.0	-26.7 to -23.2	
PiMS, N = 179	-36.8	-40.8 to -32.7	0.43	-24.1	-29.5 to -18.6	0.75
PiMZ, N = 52	-35.4	-42.8 to -27.9	0.94	-21.6	-31.6 to -11.6	0.51
**Persistent Smokers** b						
PiMM, N = 922	-35.6	-39.3 to -31.9		-26.6	-31.3 to -21.9	
PiMS, N = 74	-41.7	-48.7 to -34.8	0.07	-30.6	-39.4 to -21.7	0.35
PiMZ, N = 18	-41.2	-54.5 to -27.9	0.40	-24.0	-40.8 to -7.1	0.75
**Obese subjects** c						
PiMM, N = 653	-36.8	-42.4 to -31.3		-33.6	-41.1 to -26.1	
PiMS, N = 55	-37.1	-46.3 to -28.0	0.94	-29.7	-42.1 to -17.4	0.48
PiMZ, N = 16	-48.0	-62.9 to -33.1	0.12	-34.7	-54.8 to -14.6	0.91

CI: confidence interval. FEV1: forced expiratory volume in 1 second. FVC: forced vital capacity.

Covariates included sex, linear and squared age, recruiting area, smoking history (packyears at baseline, as well as linear and squared packyears between baseline and follow-up), height, baseline BMI and BMI change between baseline and follow-up.

a Passive smokers were defined as never smokers who declared regular exposure to environmental tobacco smoke within one year prior to the baseline or follow-up examination.

b Persistent smokers were classified as subjects who declared current smoking at both examinations.

c Obese subjects were defined as BMI≥30kg/m^2^ at the baseline or follow-up examination.

**Table 3:** Adjusted mean values in **Δ** (FEV1/FVC) and **Δ**FEF25-75% over 11 years of follow-up comparing different *SERPINA1* genotypes.

	ΔFEF25-75%(ml/y)	95%CI	P-value	Δ (FEV1/FVC)(‰/y)	95%CI	P-value
**Total**						
PiMM, N = 4207	-70.6	-72.5 to -68.7		-4.03	-4.17 to -3.89	
PiMS, N = 356	-74.4	-80.8 to -68.0	0.26	-4.14	-4.62 to -3.66	0.65
PiMZ, N = 112	-81.4	-92.8 to -70.0	0.07	-4.46	-5.31 to -3.60	0.33
**Passive Smokers** a						
PiMM, N = 691	-67.5	-72.4 to -62.5		-3.88	-4.23 to -3.53	
PiMS, N = 52	-65.5	-82.8 to -48.3	0.84	-3.01	-4.23 to -1.79	0.18
PiMZ, N = 22	-65.8	-92.3 to -39.4	0.91	-3.11	-4.98 to -1.24	0.43
**Ever Smokers**						
PiMM, N = 2194	-70.0	-73.0 to -67.1		-3.84	-4.06 to -3.62	
PiMS, N = 179	-74.9	-84.2 to -65.6	0.32	-4.16	-4.85 to -3.47	0.38
PiMZ, N = 52	-87.4	-104.5 to -70.4	0.05	-4.80	-6.07 to -3.53	0.14
**Persistent Smokers** b						
PiMM, N = 922	-66.8	-75.2 to -58.4		-3.90	-4.53 to -3.27	
PiMS, N = 74	-74.0	-89.8 to -58.3	0.34	-4.62	-5.81 to -3.44	0.21
PiMZ, N = 18	-108.2	-138.1 to -78.2	0.005	-5.25	-7.51 to -2.99	0.23
**Obese Subjects** c						
PiMM, N = 653	-58.4	-70.1 to -46.7		-2.81	-3.70 to -1.93	
PiMS, N = 55	-59.5	-78.8 to -40.1	0.90	-3.61	-5.07 to -2.15	0.22
PiMZ, N = 16	-92.2	-123.7 to -60.6	0.03	-5.13	-7.52 to -2.75	0.05
**Subjects in upper tertile of hs-CRP** d						
PiMM, N = 1387	-71.2	-74.8 to -67.6		-4.00	-4.28 to -3.71	
PiMS, N = 99	-89.8	-102.0 to -77.7	0.003	-5.03	-5.99 to -4.06	0.04
PiMZ, N = 36	-99.3	-119.3 to -79.4	0.006	-5.46	-7.04 to -3.88	0.07

CI: confidence interval. FEF25-75%: forced mid expiratory flow. FEV1: forced expiratory volume in 1 second. FVC: forced vital capacity.

Covariates included sex, linear and squared age, recruiting area, smoking history (packyears at baseline, as well as linear and squared packyears between baseline and follow-up), height, baseline BMI and BMI change between baseline and follow-up.

a Passive smokers were defined as never smokers who declared regular exposure to environmental tobacco smoke within one year prior to the baseline or follow-up examination.

b Persistent smokers were classified as subjects who declared current smoking at both examinations.

c Obese subjects were defined as BMI≥30kg/m^2^ at the baseline or follow-up examination.

d Corresponded to a level of ≥ 1.8 mg/l.

High-sensitivity C-reactive Protein (hs-CRP) as a Proxy for Low Systemic Inflammation

In order to strengthen the hypothesis that low grade systemic inflammation drove the observed Z allele effect on pulmonary function, we investigated the associations in participants with elevated hs-CRP levels at follow-up (defined as the upper tertile of the study population, i.e.≥1.8 mg/l). Consistent with our findings for smokers and obese people, declines in PiZ heterozygotes were significantly enhanced for FEF25-75% (99.3 ml (PiMZ) vs. 71.2 ml (PiMM), p = 0.006) and suggestively for FEV1/FVC (5.46‰ (PiMZ) vs. 4.00‰ (PiMM), p = 0.07) (Table 3), but not for FEV1 or FVC. Interestingly, also PiS heterozygotes in the upper tertile of hs-CRP values showed statistically significant larger declines in FEF25-75% (p = 0.003) and FEV1/FVC (p = 0.04) than PiMM subjects.

### Incidence of Airway Obstruction and Respiratory Symptoms

As a next step we tried to expand our analyses on the change of spirometry measures with clinically relevant outcomes like the incidence of airway obstruction or the development of respiratory symptoms. 103 PiMZ carriers did not show airway obstruction at baseline which was defined by FEV1/FVC < 0.7. In 17 of them a smaller ratio than 0.7 was calculated at follow-up, which classified them as incident cases. Comparing with PiMM individuals this resulted in an adjusted odds ratio of 1.11 (95% CI = 0.64 to 1.93, p = 0.70; Table 4). Corresponding investigations with individuals exposed to elevated inflammatory conditions did not show an increased obstruction risk for Z heterozygotes, but were based on very few numbers of cases. As far as the development of respiratory symptoms like regular cough, phlegm or shortness of breath during sleep was concerned, there seemed to be a trend that PiMZ individuals were more susceptible than PiMM carriers, in particular in subgroups exposed to pro-inflammatory conditions (Table 4). But the limited statistical power did unfortunately not allow a comprehensive evaluation of our hypothesis.

**Table 4:** Adjusted odds ratios for developing airway obstruction (FEV1/FVC<0.7) and respiratory symptoms over 11 years of follow-up comparing different *SERPINA1* genotypes.

	Incidence of Airway Obstruction				Incidence of Respiratory Symptoms			
	N (cases/total)	OR	95%CI	P-value	N (cases/total)	OR	95%CI	P-value
**Total**								
PiMM	601/3775	ref.			380/3547	ref.		
PiMS	50/315	0.97	0.70 to 1.35	0.86	33/307	1.07	0.73 to 1.57	0.73
PiMZ	17/103	1.11	0.64 to 1.93	0.70	15/101	1.52	0.86 to 2.69	0.15
**Ever Smokers**								
PiMM	346/1920	ref.			240/1780	ref.		
PiMS	30/152	1.12	0.72 to 1.74	0.62	18/144	0.99	0.58 to 1.66	0.96
PiMZ	9/47	1.20	0.56 to 2.60	0.64	10/43	1.94	0.93 to 4.06	0.08
**Persistent Smokers** a								
PiMM	162/801	ref.			131/705	ref.		
PiMS	14/63	0.90	0.46 to 1.78	0.76	8/56	0.76	0.34 to 1.68	0.50
PiMZ	1/17	0.29	0.04 to 2.33	0.25	3/13	1.19	0.31 to 4.56	0.80
**Obese Subjects** b								
PiMM	94/555	ref.			60/514	ref.		
PiMS	6/47	0.63	0.25 to 1.62	0.34	6/43	1.26	0.49 to 3.22	0.63
PiMZ	4/15	1.98	0.57 to 6.83	0.28	4/16	3.24	0.95 to 11.07	0.06
**Subjects in upper tertile of hs-CRP** c								
PiMM	229/1200	ref.			140/1122	ref.		
PiMS	18/88	1.01	0.57 to 1.79	0.96	13/84	1.35	0.71 to 2.56	0.35
PiMZ	7/34	1.04	0.42 to 2.55	0.93	8/32	2.51	1.07 to 5.85	0.03

CI: confidence interval. hs-CRP: high-sensitivity C-reactive protein. OR: odds ratio.

Covariates included sex, linear and squared age, recruiting area, smoking history (packyears at baseline, as well as linear and squared packyears between baseline and follow-up), height, baseline BMI and BMI change between baseline and follow-up.

a Persistent smokers were classified as subjects who declared current smoking at both examinations.

b Obese subjects were defined as BMI≥30kg/m^2^ at the baseline or follow-up examination.

c Corresponded to a level of ≥ 1.8 mg/l.

### Validity

As chronic asthma is suggested to be associated with accelerated loss of pulmonary function [16] and in the absence of post-bronchodilation spirometry, we conducted a sensitivity analysis by including only subjects who did not report physician-diagnosed asthma. Furthermore, level of lung function change in adults is also determined by lung function growth during early adulthood, and to account for that, we performed another sensitivity analysis by only including participants older than 30 years of age at baseline. Neither of the two restrictions did essentially alter the results (Table S1).

Replacing the change of FEF25-75% by the change of the ratio FEF25-75%/FVC led to very similar conclusions (Table S2). Finally, in order to detect potential participation bias, we weighted each observation inverse to the probability of being included in the study sample. None of the results of the regression analyses did materially change (Table S3).

## Discussion

In the present study, neither PiS nor PiZ heterozygosity influenced longitudinal lung function measured by **Δ**FEV1 or **Δ**FVC, independent of smoking or obesity status. However, PiMZ genotype was associated with an accelerated FEF25-75% decline in smoking and obesity subgroups from the general population. Results for participants in the upper tertile of hs-CRP values strengthened the notion that PiMZ carriers might be more susceptible to systemic pro-inflammatory conditions with respect to lung function parameters indicating narrowing of small airways.

### The Role of Smoking and Pulmonary Oxidative Stress

It is well established that smokers suffering from severe AAT deficiency, a condition accompanied by only 15% of normal AAT blood concentrations, are particularly vulnerable to developing early onset COPD [2]. There is less evidence that intermediate or even mild AAT deficiency modify the effect of smoking or other oxidative inhalants on lung function. In a small study of 56-year-old men, a higher mean annual decrease in FEV1 in smoking PiMZ individuals was reported as compared with non-smoking PiMZ or smoking PiMM individuals [17], but larger studies could not find such an interaction [7], [9]. Results of studies investigating the impact of environmental and occupational exposure are heterogeneous [18]. Passive smoking has been shown in school children to be associated with cross-sectional lower lung function only in children having low levels of AAT in the blood, and particularly in measures of mid- to end-expiratory flow rates [19]. A recent longitudinal study found accelerated lung function declines in New York City firefighters of PiMZ genotype compared to PiMM in the years after World Trade Center collapse accompanied by massive air pollution, and no such difference could be observed prior to September 11, 2001 [20].

Support for a gene-environment interaction comes also from genome-wide as well as from experimental studies. While genome-wide association studies on COPD and lung function have not found the *SERPINA1* locus among the top hit signals [21], [22], [23], it was the most strongly associated candidate gene in ever smokers in a comprehensive evaluation of potential lung function associated genes in more than 20,000 individuals from the general population [24]. Oxidation of the Z form by cigarette smoke induced its polymerization in lung tissue of transgenic PiZZ mice [12]. Such polymers could also be detected on the lung surface in human PiZ homozygotes, originating either from lung epithelial cells or macrophages and independent of the main hepatocytic secretion [25]. In addition to being an ineffective elastase inhibitor, these polymers attracted a higher number of neutrophils, further shifting the protease-antiprotease equilibrium towards proteolysis. As subjects with PiMZ genotype also exhibit elevated levels of intrapulmonary neutrophilic inflammation [26], they are potentially more susceptible to oxidative inhalants. The observed dose-response trend in the interaction between smoking and the presence of a Z allele is in agreement with this biological concept.

### The Role of Obesity and Systemic Low Grade Inflammation

The observed accelerated loss of pulmonary function in obese subjects carrying the PiMZ genotype is novel. The often observed inverse association between obesity and lung function is in part explained by a mechanical effect of obesity on lung volume and airway caliber. Yet more limited evidence suggests an additional effect of obesity on peripheral airway obstruction that may be related to systemic low grade inflammation [27]. Adipose tissue from subjects who are overweight or obese produces pro-inflammatory adipokines that spill over to the blood stream. Elevated circulating concentrations of inflammation markers such as hs-CRP or interleukin-6 (IL-6) are both, higher in obese persons [28], [29] and associated with accelerated lung function decline [30], [31]. Also in the SAPALDIA cohort, we have previously reported that accelerated lung function decline and obesity are both associated with increased hs-CRP, particularly in women [32]. Mendelian randomization approaches to examine causality between increased hs-CRP and lower respiratory function led to conflicting results [30], [33]. However, these studies did not assess the effect of *CRP* gene variants on lung function separately in groups exposed to pro-inflammatory agents such as smokers.

### This Study in Context

A longitudinal study in smokers reported an overrepresentation of PiMZ participants in rapid lung function decliners [10]. However, population-based cohort studies did not find consistent PiMZ effects on lung function decline, neither generally, nor in smoking strata [7], [8], [9]. The only marginally statistical significant result was found in non-smoking PiMZ carriers who showed a steeper unadjusted lung function decline than non-smoking PiMM subjects [7]. Our study was generally in good agreement with these results as we could not detect any statistical significant effect of *SERPINA1* genotypes on **Δ**FEV1, which was the primary focus of the previously mentioned studies. Compared to those existing publications we included in our analysis a wider range of spirometry measures and found associations with the decline of FEF25-75% as well as weaker, but relatively consistent associations with the decline of the FEV1/FVC ratio. Both these two measures are in use to assess early airway obstruction and may probably best reflect the volume of the small airways [34], [35]. There are hardly any large studies which used these measures in connection with *SERPINA1* genotypes so far, apart from a recent study of two large populations that found PiMZ genotypes associated with lower FEV1/FVC ratio and with more severe emphysema on chest computer tomography scan, but not with COPD status [36]. Flow related spirometric characteristics such as FEF25-75% may be decreased in the presence of airway abnormalities including inflammation or alterations in elastic recoil, two important correlates of AAT deficiency [37]. For example, interactions between glutathione S-transferase (GST) deficiency genotypes and passive smoking were strongest for mid expiratory flow measures in children [38]. However, these measures have often been criticized for being more variable and therefore less reliable than FEV1 [39]. We observed a correlation coefficient of 0.82 between baseline and follow-up FEF25-75% in SAPALDIA which is smaller than the one for FEV1 (0.92) and FVC (0.91), but larger than the one for the more commonly used FEV1/FVC ratio (0.74). Since we consistently found main and interacting effects of air pollution strongest for this mid flow parameter [40], [41], [42], it seems unlikely that the results of the present study are driven by measurement error. Moreover, FEF25-75% was found to have a high heritability in families with severe COPD [37].

### Strengths and Weaknesses

The strength of this study is its large sample size, its detailed characterization of subjects, and its stringent quality control of spirometry [43]. The credibility of our results is supported by the fact that the reduction of AAT serum levels in PiMS and PiMZ compared to PiMM subjects was similar to that described by others [7]. Compared to the hitherto existing publications, we carefully excluded carriers of additional, rare mutations influencing AAT serum levels in order to diminish misclassification of wildtype alleles.

Our study has some limitations. First, a possible selection of healthy individuals may limit the generalizability of the results. However, giving more weight to underrepresented groups within the study sample did not alter the results. Moreover, if persons with low levels of lung function were preferentially lost among PiMZ carriers, stated effects may be an underestimation of the true effect. Second, PiMZ individuals showed slightly higher baseline FEF25-75% and FEV1/FVC values which can be partially explained by the younger age and the reduced number of smokers in this group, but which may question the clinical relevance of the accelerated decline in these measures. Yet, the combination of a higher level of cross-sectional lung function and a steeper lung function decline after exposure to inflammatory agents parallels observations in New York City firefighters before and after the September 11 attacks [20]. Furthermore, spirometric measurements were carried out without bronchodilator [44], which hinders a clinically acceptable definition of airway obstruction. Unlike some comparable studies [9], [10], we performed spirometry at only two time points. This makes our change values susceptible to imprecision, but we do not expect substantial measurement error for several reasons. The same spirometers and stringent quality criteria at baseline and follow-up were applied, correlation coefficients between the measurements at the two time points were high, and the direction of most genotype effects were consistent for all lung function outcomes. In addition, since it would be unlikely that any measurement error is associated with the *SERPINA1* genotype, misclassification would be non-differential, which indicates an underestimation rather than an overestimation of the real effect. Another limitation is the low statistical power for analyzing obese or persistent smoking PiMZ carriers despite the large cohort size. Therefore, our findings must be interpreted with caution. If a correction for multiple testing was applied, none of the results would remain statistically significant (since all observed p-values > 0.001). For the same reason, we could neither distinguish emerging from persistent obesity, nor could we form strata with enhanced hs-CRP levels of clinical relevance (i.e.>10 mg/l). Finally, the clinical meaning of the observed excess decline in PiZ heterozygotes could not be reliably estimated, as the numbers of incident cases with airway obstruction or respiratory symptoms were too small to investigate differences between *SERPINA1* genotype classes with respect to inflammatory conditions. Nevertheless, we found indication that those subgroups are more likely to develop respiratory symptoms and we know from literature that mid expiratory flow rates have been shown to be a powerful predictor of mortality from COPD, independent of FEV1 [45].

## Conclusion

We confirm in this population-based study that neither PiMS, nor PiMZ carriers have a substantial impact on longitudinal lung function. There is indication however, that the presence of one Z allele may be sufficient to accelerate loss of small airway volume and incidence of respiratory symptoms in defined population subgroups which are exposed to pro-inflammatory agents and conditions. This is a potentially relevant observation as the prevalence of PiMZ genotype is quite common in Western Europe [3] and was 2.4% in this study sample. In order to estimate the public health relevance of our findings, future studies must associate subgroups of PiMZ individuals to post-bronchodilator lung function and clinically relevant outcomes like respiratory symptoms, emphysema by chest computer tomography and hospitalization for COPD.

## Materials and Methods

### Ethics Statement

SAPALDIA was approved by the Swiss Academy of Medical Sciences, the supraregional ethics committee for clinical research (UREK, Project Approval Number 123/00) and the Cantonal Ethics Committees for each of the eight examination areas (Ethics commissions of the cantons Aargau, Basel, Geneva, Grisons, Ticino, Valais, Vaud and Zurich). Participants were required to give written consent before any part of the health examination was conducted either globally (for all health examinations) or separately for each investigation.

### Study Population

In 1991, a random sample of 9651 adults, aged 18-60 years, from eight areas in Switzerland underwent a detailed health examination including a questionnaire about respiratory health, occupational and lifestyle exposures [46]. Participants were predominantly of European-Caucasian ethnicity and represented urban and rural areas. Eleven years later, 8047 persons were reassessed [47]. 6058 follow-up subjects provided blood samples and consented to DNA analysis. 5274 of these subjects underwent spirometry testing at baseline and follow-up. Not included in this analysis were participants with missing smoking history or body mass index (BMI) data (n = 525), subjects without valid hs-CRP (n = 18), and subjects for whom genotyping of the S or Z allele either failed (n = 6) or resulted in PiS homozygosity (n = 10), PiSZ compound heterozygosity (n = 10) or PiZ homozygosity (n = 1). Other *SERPINA1* rare mutations which lower AAT blood levels were detected according to a procedure described elsewhere [48] in additional 29 samples which were also excluded. Our study sample included thus 4675 subjects.

### Measurements

Spirometry was assessed according to American Thoracic Society criteria using the same spirometers in 1991 and 2002 (Sensormedics model 2200, USA) and by applying stringent quality control criteria [43]. The forced expiratory manoeuvre was obtained without bronchodilators. FEV1 and FVC had to originate from the same manoeuvre in order to provide a valid FEV1/FVC ratio. Information about the smoking history was collected by questionnaire. Passive smoking was positive if never smoking subjects gave an affirmative answer at baseline or follow-up to the question if they were exposed to environmental tobacco smoke in the 12 months prior to the examination on most days or nights. Height and weight were measured and BMI was calculated as weight divided by squared height. Incident cases of airflow obstruction were defined as persons with a FEV1/FVC ratio ≥ 0.7 at baseline and < 0.7 at follow-up and were compared to individuals without obstruction at both examinations. Incident cases of respiratory symptoms were defined as people with self-reported regular cough, phlegm or shortness of breath at follow-up, but not at baseline. They were compared with individuals without any of these symptoms at baseline and follow-up. Cough or phlegm had to be present during the day or at night on most days for as much as 3 months per year and shortness of breath had to occur during sleep in the past 12 months before the examination. Subjects who declared an asthma diagnosis by a physician at baseline or follow-up were defined as asthmatics.

### Serum Analysis

AAT and hs-CRP concentrations were determined from blood serum aliquots by latex-enhanced immunoturbidimetric assays (Roche diagnostics, Germany). Lower detection thresholds for the AAT and CRP assays were 0.21 g/l and 0.1 mg/l, respectively.

### Genotyping

Genotyping of *SERPINA1* PiS (rs17580) and PiZ (rs28929474) polymorphisms was carried out using 5′ nuclease fluorescent real-time PCR (TaqMan Probes technology) on LightCycler480 (Roche) as described before [4]. Probes and primers are given in Table S4. Genotype distributions for PiS and PiZ were both in Hardy Weinberg equilibrium (p = 0.93, N = 6050, and p = 0.99, N = 6051, respectively).

### Statistical Analysis

Statistical tests to evaluate differences in the characteristics among the different groups of genotype carriers encompassed Pearson's χ^2^ for testing equal proportions, analysis of variance (ANOVA) for testing equal means of normally distributed continuous data, and Kruskal-Wallis for testing equal distributions of continuous data which were not normally distributed. Main effects of *SERPINA1* alleles on lung function decline were assessed using multiple unconditional linear regression models adjusted for sex, age, recruiting area, smoking history (packyears at baseline and between baseline and follow-up), height, baseline BMI and BMI change between baseline and follow-up. Age and packyears between baseline and follow-up were modeled with linear and squared terms to better fit to spirometry data. Interactions between genotypes and other covariates were tested by integrating multiplicative terms in the regression models. Two-sided p-values of <0.05 (and of <0.10 for interactions) were considered as statistically significant. We performed 56 different linear regression tests (4 respiratory outcomes * 2 genotype comparisons * 7 categories). Bonferroni correction would thus lower the significance threshold to p = 0.05/56 = 0.001. However, since all analyses were hypothesis-driven and most tests not independent of each other, we decided to give the results uncorrected for multiple testing.

Logistic regression models were used to compare the odds of developing airflow obstruction or respiratory symptoms between baseline and follow-up among the *SERPINA1* genotype classes. The models were adjusted for the same covariates mentioned above. All statistical analyses were performed with STATA, release 10.1 IC (STATA corporation, USA).

## Supporting Information

Table S1 **Sensitivity analyses for adjusted mean values in Δ(FEV1/FVC) and ΔFEF25-75% over 11 years of follow-up comparing different** ***SERPINA1*** **genotypes.**
(PDF)

Table S2 **Adjusted mean values in Δ (FEF25-75%/FVC) over 11 years of follow-up comparing different** ***SERPINA1*** **genotypes.**
(PDF)

Table S3 **Adjusted mean values in lung function change over 11 years of follow-up comparing different** ***SERPINA1*** **genotypes in unweighted and weighted models.**
(PDF)

Table S4 **Primers and probes for genotyping the** ***SERPINA1*** **PiS and PiZ polymorphisms (rs17580 and rs28929474) using 5′ nuclease fluorescent real-time PCR (TaqMan Probes technology) on LightCycler480 (Roche).**
(PDF)
