# Supplementary material for: SERPINA1 PiZ and PiS Heterozygotes and Lung Function Decline in the SAPALDIA Cohort
Source: PLoS One. 2012 Aug 13;7(8):e42728. doi: 10.1371/journal.pone.0042728 (PMC3418297; doi:10.1371/journal.pone.0042728)
Supplement: Table S1 — Sensitivity analyses for adjusted mean values in Δ(FEV1/FVC) and ΔFEF25-75% over 11 years of follow-up comparing different SERPINA1 genotypes. (PDF) [file pone.0042728.s001.pdf]

Table S1. Sensitivity analyses for adjusted mean values in Δ(FEV1/FVC) and ΔFEF25-75% over 11 years of follow-up comparing different *SERPINA1* genotypes.

All	n	ΔFEF25-75%(ml/y)	p-value	Δ(FEV/FVC) (‰/y)	p-value
MM	4207	-70.60		-4.03	
MS	356	-74.42	0.26	-4.14	0.65
MZ	112	-81.41	0.07	-4.46	0.33
MM, age>30	3320	-70.93		-3.84	
MS, age>30	291	-75.20	0.25	-3.97	0.67
MZ, age>30	90	-84.42	0.04	-4.47	0.21
MM, non-asthmatics	3751	-71.41		-3.99	
MS, non-asthmatics	316	-73.17	0.63	-3.95	0.90
MZ, non-asthmatics	102	-80.82	0.13	-4.39	0.38
Ever smokers					
MM	2194	-70.02		-3.84	
MS	179	-74.91	0.32	-4.16	0.38
MZ	52	-87.44	0.05	-4.80	0.14
MM, age>30	1800	-69.12		-3.78	
MS, age>30	156	-73.48	0.41	-3.92	0.71
MZ, age>30	44	-86.62	0.07	-4.65	0.22
MM, non-asthmatics	1957	-70.52		-3.78	
MS, non-asthmatics	156	-71.52	0.85	-3.84	0.86
MZ, non-asthmatics	46	-86.07	0.10	-4.74	0.16
Persistent smokers					
MM	922	-66.82		-3.90	
MS	74	-74.02	0.34	-4.62	0.21
MZ	18	-108.16	0.005	-5.25	0.23
MM, age>30	708	-66.63		-4.03	
MS, age>30	62	-75.62	0.27	-4.61	0.37

MZ, age>30	12	-105.47	0.03	-4.54	0.72
MM, non-asthmatics	844	-66.81		-3.77	
MS, non-asthmatics	64	-68.99	0.79	-4.08	0.62
MZ, non-asthmatics	18	-107.33	0.007	-5.09	0.24
Obese Subjects					
MM	653	-58.39		-2.81	
MS	55	-59.46	0.90	-3.61	0.22
MZ	16	-92.16	0.03	-5.13	0.05
MM, age>30	581	-68.14		-3.19	
MS, age>30	51	-64.06	0.64	-3.56	0.59
MZ, age>30	16	-99.22	0.04	-5.49	0.05
MM, non-asthmatics	561	-59.56		-2.75	
MS, non-asthmatics	47	-58.67	0.92	-3.38	0.36
MZ, non-asthmatics	14	-95.23	0.03	-5.32	0.04
Subjects in upper tertile of hs-CRP					
MM	1387	-71.24		-4.00	
MS	99	-89.85	0.003	-5.03	0.04
MZ	36	-99.32	0.006	-5.46	0.07
MM, age>30	1174	-70.80		-3.98	
MS, age>30	86	-89.02	0.006	-5.00	0.06
MZ, age>30	31	-94.91	0.03	-5.54	0.08
MM, non-asthmatics	1229	-71.47		-3.86	
MS, non-asthmatics	86	-87.06	0.02	-4.55	0.20
MZ, non-asthmatics	33	-101.16	0.006	-5.62	0.04

Covariates included sex, linear and squared age, recruiting area, smoking history (packyears at baseline, as well as linear and squared packyears between baseline and follow-up), height, baseline BMI and BMI change between baseline and follow-up.
Persistent smokers were classified as subjects who declared current smoking at both examinations.
Obese subjects were defined as BMI ≥ 30kg/m^2 at the baseline or follow-up examination.
Subjects in the upper tertile of hs-CRP had blood levels of ≥ 1.8 mg/l.
