# Supplementary material for: SERPINA1 PiZ and PiS Heterozygotes and Lung Function Decline in the SAPALDIA Cohort
Source: PLoS One. 2012 Aug 13;7(8):e42728. doi: 10.1371/journal.pone.0042728 (PMC3418297; doi:10.1371/journal.pone.0042728)
Supplement: Table S2 — Adjusted mean values in Δ (FEF25-75%/FVC) over 11 years of follow-up comparing different SERPINA1 genotypes. (PDF) [file pone.0042728.s002.pdf]

Table S2. Adjusted mean values in Δ(FEF25-75%/FVC) over 11 years of follow-up comparing different *SERPINA1* genotypes.

All	Δ(FEF25-75%/FVC) (‰/y)	95%CI	p-value
MM, n=4207	-13.09	-13.56 to -12.62	
MS, n=356	-13.65	-15.28 to -12.01	0.52
MZ, n=112	-15.80	-18.70 to -12.89	0.07
Ever smokers			
MM, n=2194	-12.77	-13.51 to -12.04	
MS, n=179	-13.75	-16.05 to -11.44	0.42
MZ, n=52	-17.37	-21.59 to -13.15	0.03
Persistent smokers			
MM, n=922	-12.50	-14.57 to -10.43	
MS, n=74	-13.68	-17.55 to -9.82	0.52
MZ, n=18	-21.58	-28.94 to -14.22	0.01
Obese subjects			
MM, n=653	-9.33	-12.45 to -6.21	
MS, n=55	-10.01	-15.16 to -4.87	0.76
MZ, n=16	-17.67	-26.07 to -9.28	0.04
Subjects in upper tertile of hs-CRP			
MM, n=1387	-13.05	-14.02 to -12.07	
MS, n=99	-16.77	-20.07 to -13.47	0.03
MZ, n=36	-20.80	-26.21 to -15.38	0.006

Covariates included sex, linear and squared age, recruiting area, smoking history (packyears at baseline, as well as linear and squared packyears between baseline and follow-up), height, baseline BMI and BMI change between baseline and follow-up.
Persistent smokers were classified as subjects who declared current smoking at both examinations.
Obese subjects were defined as BMI ≥ $30kg/m^2$ at the baseline or follow-up examination.
Subjects in the upper tertile of hs-CRP had blood levels of ≥ 1.8 mg/l.
