# Supplementary material for: SERPINA1 PiZ and PiS Heterozygotes and Lung Function Decline in the SAPALDIA Cohort
Source: PLoS One. 2012 Aug 13;7(8):e42728. doi: 10.1371/journal.pone.0042728 (PMC3418297; doi:10.1371/journal.pone.0042728)
Supplement: Table S3 — Adjusted mean values in lung function change over 11 years of follow-up comparing different SERPINA1 genotypes in unweighted and weighted models. (PDF) [file pone.0042728.s003.pdf]

Table S3. Adjusted mean values in lung function change over 11 years of follow-up comparing different *SERPINA1* genotypes in unweighted and weighted models.

All	n	ΔFEV1(ml/y)	p-value	ΔFVC(ml/y)	p-value	ΔFEF25-75%(ml/y)	p-value	Δ(FEV/FVC) (‰/y)	p-value
MM	4207	-35.16		-24.11		-70.60		-4.03	
MS	356	-36.91	0.24	-24.68	0.77	-74.42	0.26	-4.14	0.65
MZ	112	-36.02	0.74	-23.27	0.80	-81.41	0.07	-4.46	0.33
MM, weighted	4207	-35.15		-24.14		-70.57		-4.02	
MS, weighted	356	-36.88	0.25	-24.55	0.84	-74.49	0.24	-4.16	0.60
MZ, weighted	112	-36.21	0.68	-23.64	0.89	-81.61	0.10	-4.47	0.29

The weights were calculated inverse to the probability of being included in the study sample (based on age, sex and recruiting area variables).
