# Supplementary material for: SERPINA1 PiZ and PiS Heterozygotes and Lung Function Decline in the SAPALDIA Cohort
Source: PLoS One. 2012 Aug 13;7(8):e42728. doi: 10.1371/journal.pone.0042728 (PMC3418297; doi:10.1371/journal.pone.0042728)
Supplement: Table S4 — Primers and probes for genotyping the SERPINA1 PiS and PiZ polymorphisms (rs17580 and rs28929474) using 5′ nuclease fluorescent real-time PCR (TaqMan Probes technology) on LightCycler480 (Roche). (PDF) [file pone.0042728.s004.pdf]

Table S4. Primers and probes for genotyping the *SERPINA1* PiS and PiZ polymorphisms (rs17580 and rs28929474) using 5’ nuclease fluorescent real-time PCR (TaqMan Probes technology) on LightCycler480 (Roche).

	**PiS (rs17580)**	**PiZ (rs28929474)**
Primer Forward	5'-GCCATCTTCTTCCTGCCTGAT-3'	5'-TCCAAGGCCGTGCATAAGG-3'
Primer Reverse	5'-CCAGGAACTTGGTGATGATATCGT-3'	5'-GCCCCAGCAGCTTCAGT-3'
VIC dye-labeled probe	5'-CACCTGGAAAATGAA-3'	5'-ACCATCGACGAGAAAG-3'
FAM dye-labeled probe	5'-CACCTGGTAAATGAA-3'	5'-CATCGACAAGAAAG-3'
